# Does river channelization increase the abundance of invasive crayfish? Survey of *Faxonius limosus* in small Central European streams

**DOI:** 10.1007/s11356-021-12750-y

**Published:** 2021-02-22

**Authors:** Maciej Bonk, Rafał Bobrek

**Affiliations:** 1 Institute of Nature Conservation, Polish Academy of Sciences, al. Adama Mickiewicza 33, 31-120 Kraków, Poland; 2 Polish Society for the Protection of Birds, ul. Odrowąża 24, 05-270 Marki, Poland

**Keywords:** River channelization, Decapoda, Cambaridae, Habitat disturbance, River alterations, Freshwater crayfish

## Abstract

The aim of this study was to assess whether regulated river reaches show higher numbers of the spiny cheek crayfish *Faxonius limosus*, a common freshwater invader in Europe. Man-made alterations of riverine habitats may increase their susceptibility to biological invasions. This phenomenon is commonly studied in the context of dam reservoirs; however, the impact of river channelization and the resulting riverine habitat homogenization on the success of invasive species are mostly lacking. Surveys were conducted in natural and channelised river stretches in five rivers of south-central Poland. General linear mixed model analysis showed that crayfish abundance is usually higher at sites located within regulated reaches. The likely reason for the detected differences in abundance of crayfish may be related to decreased food-web complexity and lack of predators in a homogenised habitat. The results suggest that the channelization of small watercourses may increase the abundance of invasive species *F. limosus* and its pressure on native European crayfish. However, due to limited data obtained in field, our study should be considered as a pilot one.

**Supplementary Information:**

The online version contains supplementary material available at 10.1007/s11356-021-12750-y.

## Introduction

Biological invasions are a major issue for the economy (Perrings et al. 2002) and in nature conservation (McGeoch et al. 2016). Apart from applied sciences, they are also a major issue in ecological and evolutionary considerations (Mooney and Cleland 2001; Jeschke 2014). These considerations include interactions of invaders with local ecosystems, but have also been of interest in the context of colonization mechanisms in general for several decades (Hengeveld 1988). Invading species are typically ecologically flexible, highly competitive and fertile (Marchetti et al. 2004). On the other hand, successful colonization of a new area requires the habitat to be adequate for the invading species. An adequate habitat may be defined as similar to the one in the region native for the alien species, which is usually considered in terms of climate (Kriticos 2012). But local undisturbed ecosystems may be relatively resistant to invasion due to biotic ecosystem elements (Naeem et al. 2000). This resistance, however, may be affected by habitat disturbance caused by anthropogenic alteration (Byers 2002). Human alterations often lead to changes in local species composition and abundance, and thus affect ecosystem functionality (Duffy 2002), making them more susceptible to invasion (Malin et al. 2005).

In lotic freshwater ecosystems, one of the most common human-induced is river regulation. According to Ravenga et al. (Allan and Castillo 2007), most of the rivers worldwide have been, to at least some degree, affected by human activity. The regulations include dam reservoirs and other impoundments resulting in turning formerly lotic habitats into lentic ones, but also river channelization, resulting in the elimination of natural meanders, making rivers straighter and shorter than in native state. These actions lead to riverine habitat homogenization (Elosegi et al. 2010). It has been demonstrated that dam reservoirs are highly invasible (Johnson et al. 2008; Liew et al. 2016) and may act as hubs for invaders—for example, the abundance of such species decreases with increasing distance to impoundment (Light 2003). There are also many examples of the impact of river regulation on biodiversity (e.g. Poff et al. 2007; Bredenhand and Samways 2009; Clavero and Hermoso 2011). River channelization, eliminating the majority of natural hydromorphological elements, is less studied in the context of biodiversity than dam reservoirs (Aarts et al. 2004; Figarski and Kajtoch 2015). This also concerns biological invasions. Despite numerous papers documenting the invasions of alien plants along altered river channels or valleys in terrestrial habitats (e.g. Mortenson and Weisberg 2010; Catford et al. 2011), there is a lack of knowledge about the invasion process within watercourses.

The aim of the presented contribution was to test whether river channel modification, by straightening the watercourse, may facilitate the invasion of the spiny check crayfish *Faxonius limosus* (Rafinesque). Despite the presence of the species in the non-native European range encompassing the majority of the continent (Kouba et al. 2014) for more than a century, such studies have not been conducted to date. Our prediction was that in the river stretches altered by channelization, this invasive crayfish is more abundant than in natural ones*.* This contribution is important in the context of identifying riverine habitats susceptible to invasions and reducing the impact of *F. limosus* on small watercourses still inhabited by the declining noble crayfish *Astacus astacus*.

## Methods

### Study area and surveyed habitats

The studies were conducted in five small- and medium-sized rivers from the Vistula and Oder river drainage areas, located in the lower uplands and lowlands of south-central Poland. In the cases of all rivers, the bottom substrate was dominated by sand or mud, with a minority of pebbles and gravel. In one case, the Kamienna river, the regulated habitat, was dominated by stone.

Eight localities were chosen where two types of habitat (natural vs. regulated) were located directly one after the other. This resulted in eight pairs of two-habitat sections located in five rivers. Natural habitat was defined as containing natural meanders and was preselected using Google Earth maps. Consequently, a subsection without meanders visible on maps was considered regulated. To avoid classifying naturally straight subsections (which are rare in lowland and lower uplands of Poland) as regulated, straight river stretches were included only when oxbows or their remains were visible on the map or found during field observations. For each habitat in each section, 7–13 points were chosen (cross-sections) in which the following data were collected: water depth measured 50 cm from each river bank and at the central point of the river channel in each point, width of the river channel, number of points where tree roots overgrowing the bank of the watercourse and merging under water were present, percentage of macrophyte cover measured within 7–13 cross-sections as a percent of length of the cross-section covered by plants, number of pools and the number of uncovered deposits along each section. Deposits, pools and tree roots were defined according to Raven et al. (1998). While measuring the habitat features, the water temperatures were recorded in 5–13 points of each section with an accuracy of 0.1 °C.

### Crayfish sampling

Crayfish were sampled from 5 July to 21 July 2019 during low water levels. Sampling was performed within 2–3 h after dusk to decrease the effect of crayfish hiding in shelters, which could underestimate the sample. For one night, both regulated and natural habitats were surveyed in one section. In each habitat within a section, five sites, distributed evenly along the stretch, were sampled by dip netting (30 × 50 cm rectangular dip net with rake, mesh approx. 3 mm), including the substrate and macrophytes. At each sampling site, 20 full sweeps were made, always by the same person, and usually 10 sweeps for each river bank within one site. Sweeps were made ensuring the same place was not swept twice. This resulted in 100 sweeps in each section and 200 sweeps within one locality. Crayfish captured in the net were always counted by the same person. Once captured, *F. limosus* individuals were not released as it is forbidden by National law. Thus, resampling of individuals was avoided. One sampling in each habitat/section was made due to limited time and funding resources. An active survey was preferred over trapping as the pilot study in the Sanica river, with 40 baited and unbaited traps, suggested that crayfish are attracted not only by bait, but by the trap as a shelter. In shelter-poor regulated sections, the captured crayfish number could be overestimated. In fact, during two trapping sessions, the number of crayfish was always higher in the regulated section. Moreover, the frequent presence of fish in the traps, i.e., *Perca fluviatilis*, could also affect the crayfish number in the traps (M. Bonk, Bobrek R, Dołęga J. unpublished).

### Statistical analysis

A linear-mixed-effects model was used to test for differences in crayfish abundance at sites within regulated and natural subsections. The number of individuals captured at a sampling site (*N* = 80) was the response variable, the habitat type (two levels: natural and regulated) was the fixed factor and the section ID (*N* = 8), referring to the locality, was the random factor. Statistical analysis was performed using the ‘glmer’ function in the ‘lme4’ package in R 3.5.1 (2018), with Poisson distribution error. The sum of adult and juvenile individuals was included in the analysis. For the measured habitat features, the mean values with a variation coefficient were provided to describe habitat variability. Further, the differences in number of meanders × 100 m^−1^, pools × 100 m^−1^, sand deposits × 100 m^−1^, tree root sites × 100 m^−1^, mean temperatures and variation coefficients of water depth and width were tested with the Wilcoxon pairwise test for the significance of the differences between regulated and natural sections. The analyses were conducted in PAST (Hammer et al. 2001).

## Results

### River habitat description

Field observations confirmed the presumed natural or regulated character of the preselected sections. Subsections considered natural were characterised by more variable habitat features. This was reflected in a higher number of pools (*P* = 0.017) and natural meanders (*P* = 0.012; Table 1), higher values of variation coefficients of the water depth and the watercourse width (*P* = 0.017 and *P* = 0.036, respectively), usually more numerous sand depositions (*P* = 0.063; Table 1) and number of cross-sections with tree roots present (*P* = 0.025; Table 1) reflecting the usually higher tree cover along natural sections. The percentage of macrophyte cover was, on the other hand, usually higher in the regulated subsections; this was probably caused by the higher insulation of such subsections due to tree and shrub removal. Thus, this feature, to our opinion, does not correspond with the natural status of the habitat. Structures protecting the channel edges (like rip-rap) were in general not observed or appeared only sporadically within several meters of each subsection. The exception was the Kamienna river, where the regulated subsection was historically modified by stone embankments, recently destroyed and resulting in stone dominance in the bottom substrate. In the Sanica 2 and Wschodnia regulated sections, one weir was present in each section. However, along a majority of these sections, the flowing character of the watercourses was maintained. The mean temperatures for the section considered natural and regulated did not differ significantly (*P* = 0.33).

**Table 1:** Characteristics of the studied river stretches. In ‘habitat’, column N refers to natural subsections and R–to regulated subsections. Values in brackets refer to value per 100 m (for meanders, pools, sandy deposits, and tree roots and variation coefficients for mean water depth and mean river width). Symbols refers to river names as follows: K–Kamienna river, R–Radna river, S–Sanica river, Sil–Silnica river, W–Warta river, Ws–Wschodnia river

River name	Habitat	GPS^1^	Section length	Meanders (× 100 m^−1^)	Pools (× 100 m^−1^)	Sandy deposits (× 100 m^−1^)	Tree roots (× 100m^−1^)	Mean macrophyte cover	Mean water depth (var. coeff.)	Mean river width (var. coeff.)	Mean water temperature [°C]
K	N	51.10906N 20.91073E	412	3 (0.9)	5 (1.5)	7 (2.1)	8 (2.4)	0.19%	39.49 (19.45)	5.08 (0.31)	19.48
	R	51.11002N 20.87892E	331	0 (0.0)	0 (0.0)	0 (0.0)	0 (0.0)	85.50%	53.34 (11.31)	5.22 (0.087)	19.57
R1	N	50.55182N 20.96311E	569	11 (1.9)	12 (2.1)	3 (0.5)	8 (1.4)	30.93%	31.47 (4.85)	2.68 (0.05)	19.70
	R	50.56326N 20.96104E	565	2 (0.4)	0(0.0)	0 (0.0)	1 (0.2)	62.57%	37.28 (3.91)	3.98 (0.027)	18.06
R2	N	50.5431N 20.94445E	452	7 (1.5)	9 (2.0)	0 (0.0)	5 (1.1)	45.59%	54.33 (7.52)	3.93 (0.10)	22.08
	R	50.54746N 20.954E	480	0 (0.0)	1 (0.2)	0 (0.0)	1 (0.2)	79.00%	55.64 (4.19)	2.72 (0.044)	22.22
S1	N	50.4908N 20.942134E	435	5 (1.1)	13 (3.0)	17 (3.9)	5 (1.1)	12.23%	20.60 (10.47)	4.02 (0.2)	20.65
	R	50.49634N 20.92503E	397	0 (0.0)	0 (0.0)	20 (5.0)	0 (0.0)	5.90%	14.13 (0.06)	3.75 (0.06)	27.90
S2	N	50.51532N 20.88256E	259	6 (2.3)	10 (3.9)	11 (4.2)	5 (1.9)	6.77%	16.10 (3.45)	2.42 (0.049)	22.49
	R	50.51303N 20.8904E	235	0 (0.0)	0 (0.0)	5 (2.1)	6 (2.6)	13.65%	15.15 (3.67)	2.88 (0.11)	22.61
Sil	N	50.85909N 20.55139E	300	6 (2.0)	5 (1.7)	18 (6.0)	4 (1.3)	8.64%	27.7 (16.75)	3.0 (0.38)	22.08
	R	50.858498N 20.5725E	245	0 (0.0)	1 (0.4)	2 (0.8)	3 (1.2)	11%	21.6 (9.56)	3.4 (0.22)	21.50
W	N	50.73889N 19.18124E	286	3 (1.1)	4 (1.4)	2 (0.7)	6 (2.1)	26%	50.28 (13.06)	8 (0.39)	20.80
	R	50.74897N 19.18379E	400	0 (0.0)	1 (0.3)	0 (0.0)	2 (0.5)	33%	59.24 (4.87)	13.1 (0.16)	20.86
Ws	N	50.45459N 21.20616E	493	1 (0.2)	6 (1.2)	20 (4.1)	9 (1.8)	9.14%	31 (15.2)	6.25 (0.8)	21.06
	R	50.4545N 21.191968E	483	0 (0.0)	1 (0.2)	1 (0.2)	2 (0.4)	7.80%	45 (7.16)	7.45 (0.03)	22.98

### Crayfish number

**Fig. 1:**
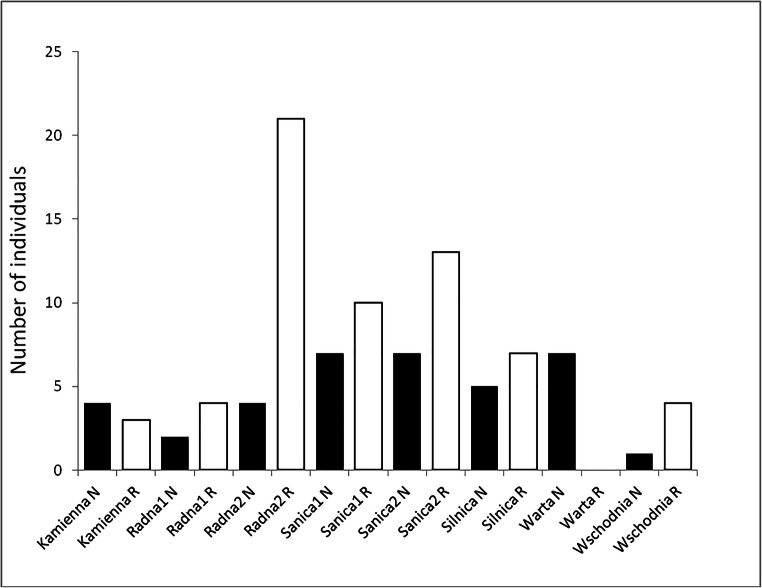
Number of *Faxonius limosus* individuals captured within the natural ‘N’ (black bars) and the regulated ‘R’ (white bars) river sections

In total, 99 individuals of *F. limosus* were captured. The number of individuals caught per sampling site was low and among-site variation was high (range from 0 to 12 per sampling site, Supp. Tab. 1). The highest number of crayfish was captured in the Sanica and Radna rivers. The mean number of individuals collected at the sampling site was 1.5 (SD = 2.42) for the regulated subsections and 0.93 (SD = 1.25) for the natural ones. The range of crayfish caught on the regulated and natural subsections (the sum of five sampling sites) ranged from 0 to 21 and from 0 to 7 individuals, respectively (Fig. 1). Usually, there were fewer individuals captured in the natural subsections, with the exceptions of the two largest rivers, where no crayfish were sampled in the regulated subsection (Warta river), or the number was slightly higher in the natural habitat (Kamienna river; Fig. 1, Suppl. Tab 1.

Generalised linear mixed model analysis showed that in general, there is a higher abundance of crayfish per site in the regulated subsections than in the natural ones (SE = 0.2055, *z* value = 2.511; *P* = 0.012; Fig. 1). However, this effect seems to be most visible in two small rivers belonging to one drainage area (Sanica and Radna rivers), whereas in the largest rivers (the Warta and the Kamienna rivers), the effect was reverse (Fig. 1).

## Discussion

Crayfish capture raises several problems. As these animals often hide in any available shelter, the number of collected individuals may be heavily underestimated. In our study, this resulted in a low number of captured individuals. Despite the number of individuals usually being higher in sites located within regulated parts of rivers, the biological significance of this relationship is unclear. In our opinion, the data should be cautiously interpreted and treated as a pilot study that warrants further investigation. Nevertheless, our results were not designed to catch the largest fraction of crayfish, but to use the same method to get comparable results in wild and channelised sections of the same river.

Despite some doubts about the accuracy of the method, our results are consistent with the general knowledge about ecosystem disturbance and its impact on biological invasions (Marvier et al. 2004; Hansen and Clevenger 2005; Elosegi et al. 2010; Case 1990; Levine and D’Antonio 1999; Naeem et al. 2000; Kennedy et al. 2002). After anthropogenic alteration of the habitat, native species may lose their superiority, gained through a long history of adaptation to the local environment (Byers 2002). This also seems to be valid in the case of our study.

However, investigating the direct factors related with river channelization, which may affect the crayfish in the studied watercourses, was not a target of our study, potential reasons for that state can be explained according to the already existing literature. The decrease in ecosystem functionality presumed for regulated subsections may be connected with the disappearance of large fish predators from the regulated river sections. Watercourse channelization affects fish diversity and composition, often reducing the abundance of trophic specialists (e.g. predators) (Aarts et al. 2004; Latli et al. 2018). This may result in lower predatory pressure on the invader. It was shown that the removal of top predators may reduce the resilience of the community to invasion of non-indigenous species (Byers 2002; Reusch 1998; Byers 2002; Ward and Ricciardi 2007). In the investigated rivers, predator elimination, overall biodiversity loss and outcompeting may presumably occur. Some anecdotal data from small rivers in Poland support the idea of a population reduction of predatory fish after river channelization (Wiśniewolski and Gierej 2011). Furthermore, it was found that the density of perch *Perca fluviatilis*, which is a common predator fish in the studied rivers, is higher in unregulated sections of rivers than in regulated ones (Bruylants et al. 1986). Furthermore, even if fish species composition is not altered, small stream channelization leads to the elimination of pools, and as a consequence, deeper microhabitats for older and larger fish (also other than perch) may disappear (Schlosser 1987; Harvey and Stewart 1991; Mallet et al. 2000; Eick 2013).

A fish survey on species composition and size structure within the studied sites was not carried out. However, to some extent, the different results in the smallest and largest rivers may support the existence of a relationship between the amount of deeper microhabitats, preferred by larger predators, and crayfish abundance. In the largest of the studied rivers (Warta and Kamienna), where both the regulated and natural subsections were deep enough (Table 1) to harbour large predatory fish, a positive effect of channelization on invasive crayfish abundance was not observed. In contrast, in the smallest and shallowest rivers (the Radna river and Sanica river), *F. limosus* was less abundant in sections characterised by a high number of pools. However, the aforementioned reverse effect (or lack of an effect) in large rivers requires further study on a larger number of natural-regulated pairs of river stretches, including broad variations in river size and depth and fish size structure as cofactors. In the case of our study, due to the low number of localities and individuals, such an analysis was not undertaken. In the examined habitats, the regulated subsections differed from the natural ones mostly by characteristics reflecting channel hydromorphology.

In general, *F. limosus* avoids small headwater streams (Chucholl 2016), which may be related to water temperature (Bonk and Bobrek 2020); thus, some of the described sections probably lie near the upper limit of the colonization ability of streams by this species. However, temperature is probably not a major reason for differences in crayfish abundance as in each locality, both regulated and unregulated sections were placed at approximately the same distance from the springs; also, the temperature in one type of section may be related to the temperature within the other due to the short distances between them. This also seems to be supported by the low differences in temperatures measured during our surveys.

*F. limosus* is a successful invader occurring in most freshwater habitat types across Europe (Holdich and Black 2006; Śmietana 2011; Kouba et al. 2014), including the brackish waters of the southern Baltic Sea (Szaniawska et al. 2017). Our study suggests that despite *F. limosus* being present in both natural and regulated river habitats, watercourse management may have an impact on its abundance and, presumably, the impact of crayfish on local ecosystems. This is important in the context of observations of several authors (Holdich and Black 2006; Chucholl 2016; Bonk and Bobrek 2020) who showed that *F. limosus* does not ingress into the smallest headwater streams, which are often a refuge of native crayfish (i.e. *Astacus astacus* is present in the Sanica and Radna rivers within the area of our study, M. Bonk, unpublished data). The channelization of such watercourses may increase the pressure of *F. limosus* on native European crayfish by transforming natural small streams into homogenised watercourses and allows its upstream dispersal. As both *F. limosus* and *A. astacus* have occurred in the studied regions in adjacent parts of streams for at least two decades, the transmission of crayfish plague is probably low or non-existent, and *A. astacus* may be competitively disadvantaged by *F. limosus* through habitat alterations. Thus, maintaining the natural character of small watercourses or renaturalising already altered streams may be helpful in maintaining native crayfish populations.

Although our results are in accordance with general knowledge about the impact of habitat disturbance on biological invasions, our data should be regarded with caution and warrant further study.

## Supplementary Information

ESM 1 (DOCX 16 kb)

## Data Availability

Provided with tables in manuscript. Raw data available in authors’ data.

## References

[CR1] Aarts BGW, Van Den Brink FWB, Nienhuis PH (2004). Habitat loss as the main cause of the slow recovery of fish faunas of regulated large rivers in Europe: the transversal floodplain gradient. River Res Appl.

[CR2] Allan DJ, Castillo MM (2007). Stream ecology.

[CR3] Bonk M, Bobrek R (2020). Will the Carpathians remain free from the spiny cheek crayfish *Faxonius limosus* (Rafinesque, 1817)?.

[CR4] Bredenhand E, Samways MJ (2009). Impact of a dam on benthic macroinvertebrates in a small river in a biodiversity hotspot: Cape Floristic Region. South Africa. J Insect Conserv.

[CR5] Bruylants B, Vandelannoote A, Verheyen R (1986). The movement pattern and density distribution of perch, *Perca fluviatilis* L., in a channelized lowland river. Aquac Res.

[CR6] Byers JE (2002). Impact of non-indigenous species on natives enhanced by anthropogenic alteration of selection regimes. Oikos.

[CR7] Case TJ (1990). Invasion resistance arises in strongly interacting species-rich model competition communities. Proc Natl Acad Sci USA.

[CR8] Catford JA, Downes BJ, Gippel CJ, Vesk PA (2011). Flow regulation reduces native plant cover and facilitates exotic invasion in riparian wetlands. J Appl Ecol.

[CR9] Chucholl C (2016). The bad and the super-bad: prioritising the threat of six invasive alien to three imperilled native crayfishes. Biol Invasions.

[CR10] Clavero M, Hermoso V (2011). Reservoirs promote the taxonomic homogenization of fish communities within river basins. Biodivers Conserv.

[CR11] Duffy JE (2002). Biodiversity and ecosystem function: the consumer connection. Oikos.

[CR12] Eick D (2013). Habitat preferences of the burbot (*Lota lota*) from the River Elbe: an experimental approach. J Appl Ichtyol.

[CR13] Elosegi A, Díez J, Mutz M (2010). Effects of hydromorphological integrity on biodiversity and functioning of river ecosystems. Hydrobiologia.

[CR14] Figarski T, Kajtoch Ł (2015). Alterations of riverine ecosystems adversely affect bird assemblages. Hydrobiologia.

[CR15] Hammer Ø, Harper DAT, Ryan PD (2001). PAST: Paleontological Statistics software package for education and data analysis. Paleontol Electron.

[CR16] Hansen MJ, Clevenger AP (2005). The influence of disturbance and habitat on the presence of non-native plant species along transport corridors. Biol Conserv.

[CR17] Harvey BC, Stewart AJ (1991). Fish size and habitat depth relationships in headwater streams. Oecologia.

[CR18] Hengeveld R (1988). Mechanisms of biological invasions. J Biogeogr.

[CR19] Holdich D, Black J (2006). The spiny-cheek crayfish, *Orconectes limosus* (Rafinesque, 1817) [Crustacea: Decapoda: Cambaridae], digs into the UK. Aquat Invasions.

[CR20] Jeschke JM (2014). General hypothesis in invasion ecology. Divers Distrib.

[CR21] Johnson PT, Olden JD, Vander Zanden MJ (2008). Dam invaders: impoundments facilitate biological invasions into freshwaters. Front Ecol Environ.

[CR22] Kennedy TA, Naeem S, Howe KM, Knops JM, Tilman D, Reich P (2002). Biodiversity as a barrier to ecological invasion. Nature.

[CR23] Kouba A, Petrusek A, Kozák P (2014). Continental-wide distribution of crayfish species in Europe: update and maps. Knowl Manag Aquat Ecosyst.

[CR24] Kriticos DJ (2012). Regional climate-matching to estimate current and future sources of biosecurity threats. Biol Invasions.

[CR25] Latli A, Michel LN, Lepoint G, Kestemont P (2018). River habitat homogenisation enhances trophic competition and promotes individual specialisation among young of the year fish. Freshw Biol.

[CR26] Levine JM, D'Antonio CM (1999). Elton revisited: a review of evidence linking diversity and invasibility. Oikos.

[CR27] Liew JH, Tan HH, Yeo DC (2016). Dammed rivers: impoundments facilitate fish invasions. Freshw Biol.

[CR28] Light T (2003). Success and failure in a lotic crayfish invasion: the roles of hydrologic variability and habitat alteration. Freshw Biol.

[CR29] Malin J, Hansen MJ, Clevenger AP (2005). The influence of disturbance and habitat on the presence of non-native plant species along transport corridors. Biol Conserv.

[CR30] Mallet JP, Lamouroux N, Sagnes P, Persat H (2000). Habitat preferences of European grayling in a medium size stream, the Ain river, France. J Fish Biol.

[CR31] Marchetti MP, Myle PB, Levin R (2004). Invasive species profiling? Exploring the characteristics of non-native fishes across invasion stages in California. Freshw Biol.

[CR32] Marvier M, Kareiva P, Neubert MG (2004). Habitat destruction, fragmentation, and disturbance promote invasion by habitat generalists in a multispecies metapopulation. Risk Anal.

[CR33] McGeoch MA, Genovesi P, Bellingham PJ, Costello MJ, McGrannachan C, Sheppard A (2016). Prioritizing species, pathways, and sites to achieve conservation targets for biological invasion. Biol Invasions.

[CR34] Mooney HA, Cleland EE (2001). The evolutionary impact of invasive species. Proc Natl Acad Sci USA.

[CR35] Mortenson SG, Weisberg PJ (2010). Does river regulation increase the dominance of invasive woody species in riparian landscapes?. Glob Ecol Biogeogr.

[CR36] Naeem S, Knops JM, Tilman D, Howe KM, Kennedy T, Gale S (2000). Plant diversity increases resistance to invasion in the absence of covarying extrinsic factors. Oikos.

[CR37] Perrings C, Williamson M, Barbier E, Delfino D, Dalmazzone S, Shogren J, Simmons P, Watkinson A (2002). Biological invasion risks and the public good: an economic perspective. Conserv Ecol.

[CR38] Poff NL, Olden JD, Merritt DM, Pepin DM (2007). Homogenization of regional river dynamics by dams and global biodiversity implications. Proc Natl Acad Sci USA.

[CR39] R Core Team (2018). R: A Language and Environment for Statistical Computing.

[CR40] Raven PJ, Holmes NTH, Dawson NH, Everard M (1998). Quality assessment using River Habitat Survey data. Aquat Conserv Mar Freshwat Ecosyst.

[CR41] Reusch TB (1998). Native predators contribute to invasion resistance to the non-indigenous bivalve *Musculista senhousia* in southern California, USA. Mar Ecol Prog Ser.

[CR42] Schlosser IJ (1987). The role of predation in age- and size-related habitat use by stream fishes. Ecology.

[CR43] Śmietana P (2011). *Orconectes limosus* (Rafinesque, 1817) In: Okarma H, Pawłowski J, Ward JM, Ricciardi A (2007) Impacts of Dreissena invasions on benthic macroinvertebrate communities: a meta-analysis. Divers Distrib.

[CR44] Szaniawska A, Dobrzycka-Krahel A, Jaszczołt J (2017). Spiny-cheek crayfish *Orconectes limosus* (Rafinesque, 1817) on its way to the open coastal waters of the Baltic Sea. Oceanol Hydrobiol St.

[CR45] Ward JM, Ricciardi A (2007) Impacts of Dreissena invasions on benthic macroinvertebrate communities: a meta-analysis. Divers Distrib 13:155–165. 10.1111/j.1472-4642.2007.00336.x

[CR46] Wiśniewolski W, Gierej A (2011) Regulacja rzek a ichtiofauna – skutki i środki zaradcze. Użytkownik Rybacki 2011- Kondycja polskiego rybactwa śródlądowego, Konferencja PZW, Spała. http://www.pzw.org.pl/pliki/prezentacje/1395/cms/szablony/11205/pliki/013_wisniewolskigierej

