# Supplementary material for: Does river channelization increase the abundance of invasive crayfish? Survey of Faxonius limosus in small Central European streams
Source: Environ Sci Pollut Res Int. 2021 Feb 22;28(24):31831–7. doi: 10.1007/s11356-021-12750-y (PMC8238746; doi:10.1007/s11356-021-12750-y)
Supplement: Supplementary file 1 — (DOCX 16 kb) [file 11356_2021_12750_MOESM1_ESM.docx]

**Supporting tab. 1.** Numbers of captured crayfish individuals in the surveyed sites with regards to habitat type (N – natural subsections, R – regulated subsections), river id and date of sampling.

| **section id** | **sample (site)** | **survey date** | **N of crayfish** | **habitat** |
| --- | --- | --- | --- | --- |
| Kamienna | 1 | 12.07.2019 | 0 | R |
| Kamienna | 2 | 12.07.2019 | 1 | R |
| Kamienna | 3 | 12.07.2019 | 2 | R |
| Kamienna | 4 | 12.07.2019 | 0 | R |
| Kamienna | 5 | 12.07.2019 | 0 | R |
| Kamienna | 1 | 12.07.2019 | 0 | N |
| Kamienna | 2 | 12.07.2019 | 0 | N |
| Kamienna | 3 | 12.07.2019 | 1 | N |
| Kamienna | 4 | 12.07.2019 | 1 | N |
| Kamienna | 5 | 12.07.2019 | 2 | N |
| Radna 1 | 1 | 13.07.2019 | 1 | R |
| Radna 1 | 2 | 13.07.2019 | 1 | R |
| Radna 1 | 3 | 13.07.2019 | 1 | R |
| Radna 1 | 4 | 13.07.2019 | 0 | R |
| Radna 1 | 5 | 13.07.2019 | 1 | R |
| Radna 1 | 1 | 13.07.2019 | 0 | N |
| Radna 1 | 2 | 13.07.2019 | 1 | N |
| Radna 1 | 3 | 13.07.2019 | 0 | N |
| Radna 1 | 4 | 13.07.2019 | 0 | N |
| Radna 1 | 5 | 13.07.2019 | 1 | N |
| Radna 2 | 1 | 9.07.2019 | 1 | R |
| Radna 2 | 2 | 9.07.2019 | 0 | R |
| Radna 2 | 3 | 9.07.2019 | 12 | R |
| Radna 2 | 4 | 9.07.2019 | 4 | R |
| Radna 2 | 5 | 9.07.2019 | 4 | R |
| Radna 2 | 1 | 9.07.2019 | 0 | N |
| Radna 2 | 2 | 9.07.2019 | 3 | N |
| Radna 2 | 3 | 9.07.2019 | 1 | N |
| Radna 2 | 4 | 9.07.2019 | 0 | N |
| Radna 2 | 5 | 9.07.2019 | 0 | N |
| Sanica 1 | 1 | 18.07.2019 | 6 | R |
| Sanica 1 | 2 | 18.07.2019 | 1 | R |
| Sanica 1 | 3 | 18.07.2019 | 0 | R |
| Sanica 1 | 4 | 18.07.2019 | 2 | R |
| Sanica 1 | 5 | 18.07.2019 | 1 | R |
| Sanica 1 | 1 | 18.07.2019 | 0 | N |
| Sanica 1 | 2 | 18.07.2019 | 5 | N |
| Sanica 1 | 3 | 18.07.2019 | 1 | N |
| Sanica 1 | 4 | 18.07.2019 | 1 | N |
| Sanica 1 | 5 | 18.07.2019 | 0 | N |
| Sanica 2 | 1 | 5.07.2019 | 2 | N |
| Sanica 2 | 2 | 5.07.2019 | 2 | N |
| Sanica 2 | 3 | 5.07.2019 | 2 | N |
| Sanica 2 | 4 | 5.07.2019 | 1 | N |
| Sanica 2 | 5 | 5.07.2019 | 0 | N |
| Sanica 2 | 1 | 5.07.2019 | 3 | R |
| Sanica 2 | 2 | 5.07.2019 | 0 | R |
| Sanica 2 | 3 | 5.07.2019 | 8 | R |
| Sanica 2 | 4 | 5.07.2019 | 1 | R |
| Sanica 2 | 5 | 5.07.2019 | 1 | R |
| Silnica | 1 | 21.07.2019 | 2 | R |
| Silnica | 2 | 21.07.2019 | 0 | R |
| Silnica | 3 | 21.07.2019 | 0 | R |
| Silnica | 4 | 21.07.2019 | 2 | R |
| Silnica | 5 | 21.07.2019 | 3 | R |
| Silnica | 1 | 21.07.2019 | 1 | N |
| Silnica | 2 | 21.07.2019 | 3 | N |
| Silnica | 3 | 21.07.2019 | 0 | N |
| Silnica | 4 | 21.07.2019 | 0 | N |
| Silnica | 5 | 21.07.2019 | 1 | N |
| Warta | 1 | 17.07.2019 | 1 | N |
| Warta | 2 | 17.07.2019 | 1 | N |
| Warta | 3 | 17.07.2019 | 0 | N |
| Warta | 4 | 17.07.2019 | 0 | N |
| Warta | 5 | 17.07.2019 | 5 | N |
| Warta | 1 | 17.07.2019 | 0 | R |
| Warta | 2 | 17.07.2019 | 0 | R |
| Warta | 3 | 17.07.2019 | 0 | R |
| Warta | 4 | 17.07.2019 | 0 | R |
| Warta | 5 | 17.07.2019 | 0 | R |
| Wschodnia | 1 | 19.07.2019 | 3 | R |
| Wschodnia | 2 | 19.07.2019 | 0 | R |
| Wschodnia | 3 | 19.07.2019 | 0 | R |
| Wschodnia | 4 | 19.07.2019 | 1 | R |
| Wschodnia | 5 | 19.07.2019 | 0 | R |
| Wschodnia | 1 | 19.07.2019 | 0 | N |
| Wschodnia | 2 | 19.07.2019 | 0 | N |
| Wschodnia | 3 | 19.07.2019 | 0 | N |
| Wschodnia | 4 | 19.07.2019 | 1 | N |
| Wschodnia | 5 | 19.07.2019 | 0 | N |
